# Case of Poisoning by Arsenic, and Chemical Examination of the Stomach

**Published:** 1850-06

**Authors:** Geo. Hadley

**Affiliations:** Professor of Chemistry and Pharmacy in the University of Buffalo


### BUFFALO MEDICAL JOURNAL

AND

### MONTHLY REVIEW.

**VOL. 6.** JUNE, 1850. **NO. 1.**

### ORIGINAL COMMUNICATIONS.

ART. I.—*Case of Poisoning by Arsenic, and Chemical Examination of the Stomach.* By Geo. Hadley, M. D., Professor of Chemistry and Pharmacy in the University of Buffalo.

The following account of the symptoms and treatment of this case is from the notes of the attending physician, Dr. J. Darling, of Smithport, Pa. It does not contain the circumstances as fully detailed as in the testimony given before the court, but is the same as far as it goes.

Of the testimony, rendered on the trial, we have very little precise information. It appeared that the defendant, Mr. Robbins, was the husband of the deceased; that he lived on bad terms with his wife; that he had purchased arsenic not long before; that he himself prepared the food, on taking which, his wife and another individual became sick, &c., &c. The jury found him guilty on the indictment for poisoning.

*Extract from Dr. Darling's Journal.*

"*Sunday, August* 5, 1849. Saw Mrs. R. 48 hours from the commencement of her illness. Appearances at that time: Pulse variable; tongue moderately coated; countenance flushed and slightly swollen; inextinguishable thirst; intense burning pain at the pit of the stomach; nausea, recurring at intervals of 10 or 15 minutes, with retchings and slight vomiting—the matter vomited has the appearance of water tinged with bile; severe pain in the head; partial fainting and difficult respiration; general restlessness; face covered with cold sweat; icy coldness, at times particularly, of the extremities, but, for the most part, hot; occasional dimness of vision; bowels costive.

Prescribed a cathartic of calomel and jalap to be followed by salts and senna, and sinapisms to the extremities and scrobiculus cordis.

*Monday morning*, Aug. 6. Cathartic operated four or five times during the night: vomiting mostly subsided; appearances otherwise somewhat aggravated—pain in the head, thirst and arterial excitement increased; performed V. S. to the extent of about 12 oz., and did not see her again until Wednesday, August 8. Countenance at that time flushed and puffy, and covered with cold, clammy sweat; tongue dry and foul; breath intolerably offensive; pulse nearly imperceptible at the wrist, and irregular; extremities permanently cold; mind delirious; great difficulty of swallowing, even of fluids; says her "throat is growing up"; hands and feet numb; vision much impaired; nails blue; breathing laborious and difficult; occasionally hickup; convulsions at frequent intervals; violent palpitation of the heart; stools dark colored and peculiarly offensive; urine scanty; picks and works at her teeth a good deal; has at times a livid, mottled appearance, and, in one or two places, a separation of the epidermis of an inch or more in extent. Died Thursday morning before daylight."

*Examination of the Stomach.*—Dr. Darling left with me, on the 13th of August, 1849, to be examined for arsenic, the stomach of the patient, whose case is described above. A little alcohol had been poured into the bottle containing it, to prevent putrefaction. The paper wrappers were taken off, and, without removing the contents, or making any examination, the bottle was carefully sealed, and, in this condition, it remained till Nov. 21, 1849.

At this date the bottle was opened and the stomach removed, and subjected to a careful examination, both as to its condition and contents. The smell was slightly putrid, like that of corpses preserved in alcohol; but putrefaction had not taken place to an extent sufficient to produce any disorganization. The alcohol, in which it was immersed, was very little changed in appearance, or discolored by any matters it had taken up. Both orifices of the stomach were tied with a black silk thread, and an inch or two of intestine remained attached to the pylorus.

On opening the stomach no particles of food could be discovered in its interior; no trace of arsenic in substance, or indeed any foreign solid matter; the only contents being about two ounces of a brown, somewhat slimy fluid, quite homogeneous in consistence. The walls of the stomach retained their ordinary appearance and texture, and presented in no part the slightest mark of lesion, with the single exception of some small, blackish spots scattered over an area of about one by one-half inches.

*Preliminary trial—Reinsch's test.*—The first trials for arsenic were made on the liquids contained in the bottle—on the alcohol, and on the fluid within the cavity of the stomach. With a view of ascertaining what might be expected in the investigation, the method of testing devised by Reinsch was tried on the latter liquid. A small portion of it was digested with dilute muriatic acid and metallic copper, and the metal afterwards heated in a small glass tube; a parallel experiment being made with the same dilute acid and the metal. The poison, however, was present in too small quantity, or the experiment was tried on too small a scale to afford any satisfactory result.

*Method Adopted, and Apparatus.*—From this trial, and from the circumstances of the case, it was inferred that the separation of the arsenic by hydrogen, or Marsh's' test, in some one or other of its modifications, would be the best, if not the only method applicable.

The gas-bottle, employed for generating the hydrogen, was constructed by taking a half pint salt-mouth and inserting into it, bottom upward, a tincture bottle, whose bottom had been cut off, of the same size, with a tube running down from it to the bottom of the first bottle; the joinings all being made air tight. In the top of the salt-mouth bottle a hole was drilled and a stopcock cemented therein. This was connected with a glass tube a few inches long, filled with dry cotton, to the end of which a capillary tube could be attached by a perforated cork. The method of operating with this apparatus will be obvious without further description. It possesses this advantage over the gas-bottles commonly employed, that it is not necessary to free the liquid tested from organic matter, because the frothy bubbles, which fill the jar, break down entirely, immediately on the concussion occasioned by the first bubble, which escapes through the tube, after the lower bottle becomes filled with gas. The hydrogen may then be drawn off through the stop-cock, being sufficiently dried by passing through the tube of cotton, and may be examined by any of the customary methods.

*Reagents, and purity of.*—The only reagents employed, in this process, were metallic zinc and dilute sulphuric acid. A few fragments of an old and very pure pig of zinc were thrown into the bottle, and some sulphuric acid, and some rain water, were set aside for the experiments. The gas cooled from these materials when burned in a jet, gave not the slightest appearance of arsenic, on bringing a cold piece of porcelain down upon the flame.

*Contents of the Stomach Examined.*—The dilute acid was then poured off, and, the same zinc remaining, a portion of the slimy liquid from the stomach substituted in its place, and sufficient acid added to produce a very moderate action. The gas now evolved burned with a flame like that of pure hydrogen. This was uniformly repeated on lighting. We are not aware that this phenomenon has been remarked before: but at this stage of the investigation no reliance was placed upon it; more especially as not the slightest stain could be obtained from the flame on white porcelain.

*By another Method.*—It was evident therefore, either that arsenic was not present, or that its quantity was so small as to require a more delicate process for its detection. The method recommended by Berzelius was adopted. To the tube filled with cotton, connected with the stopcock of the gas-bottle, a small capillary tube of hard glass containing no lead, was attached, in such a way that a portion of it could be kept red hot in the flame of a lamp. When hydrogen containing arsenic is passed through such a tube, the arsenic is separated in the metallic state, and, in consequence of its volatility, is deposited a short distance beyond the point where it is exposed to the heat.

Now on passing the gas through this tube, taking the precaution to keep it cool beyond the heated portion, no stain was observed in the first instance. After several bottles of gas, however, had been passed through it, slight spots made their appearance.

*The Alcohol tested.*—On substituting in the apparatus the pickle, in which the stomach was preserved, somewhat concentrated by evaporation, and repeating the passage of the gas through the same red hot tube, which was used in the last experiment, this deposit increased so as to form a brown ring around the tube, which, by reflected light, exhibited a slightly metallic appearance. No crystaline structure was detected in it when examined with a glass magnifying more than forty diameters.

The tube containing this crust was removed, and, in order to preserve the sublimate, was carefully sealed with the flame of the blowpipe, a short distance each side of the brown ring.

*Brown matter Oxidized.*—When heated gently over a lamp, the brown stain immediately disappeared, and, on a colder part of the tube, condensed as a white sublimate; which, by a gentle heat, could be again volatalized, and so driven along the tube condensing unchanged. So small, however, was its amount, that it was not very evident to the eye, unless it was held against a dark background; when it became perfectly distinct, and appeared like a minute white cloud lining the tube, well defined on the side where the heat had been applied, but more scattered and feeble on the other.

*Examined with a Magnifier.*—To make the nature of this sublimate more certain, it was examined with a microscope. Under a power of between 50 and 100 diameters, (the microscope is not at hand, so it is impossible to state its power exactly) it was resolved into beautiful and distinct transparent crystals, having a high degree of lustre and brilliancy. Their form was manifestly the regular octahedron.

*Not Antimony.*—All these reactions are sufficiently distinctive of arsenic. The behaviour of antimony, under the same circumstances, resembles them more nearly than that of any other substance. But, had this been the metal, the white sublimate, consisting of octahedral crystals of oxide of antimony, would not have been so readily or completely volatalizable; and the metal itself, obtained in the first instance, would have been deposited in the heated portion of the tube, and not beyond it.

*Source of the Arsenic.*—As the reagents employed in these experiments had not been subjected to a test so rigid. it remained still to prove that the source of the arsenic was not in them. To determine this point, the zinc used in the last experiments was washed several times with the acid and water, and the gas then passed, as before, through a red hot tube. All that could be obtained by continuing this process, was, not even a dark stain, but a very slight white sublimate in the place of the brown ring, or rather nearer the flame. On sealing the tube and heating as before, this was found to be readily and completely sublimable; but so small as to be seen with difficulty, and, under the same magnifying power as was used before, presented no appearance, which could be decided positively to be crystaline.

Possibly this feeble sublimate may have been arsenic: but, if it were, it might be supposed to be due to the fluids from the stomach, which, owing to the imperfect washing of the zinc, still may have adhered to the rough surfaces of its fragments.

The gas-bottle was therefore emptied and washed, some new zinc of the same description introduced, and the same acid and water employed as in the former experiments. The passage of the gas through the hot tube was pushed to a great extent, the gas-bottle being emptied above twenty times, occupying at each three to five minutes. Exceedingly feeble brownish spots now appeared, scattered along the tube for the space of one inch. Towards the close of the process a little moisture had got into the tube, which was driven out by letting more hydrogen pass through it. On treating this tube as before, there appeared, when seen under a magnifier, a sublimate as of drops of moisture, and which seemed to consist of some deliquescent substance not readily dried by opening the tube and passing dry air through it. The dark spots, which were more feeble than any seen before, were not sublimed by heat, or only with great difficulty, a temperature but little below redness not causing them entirely to disappear. This trial was not regarded as perfectly satisfactory, for the moisture obscured and rendered somewhat uncertain the result.

*Method by burning the Hydrogen.*—Another method of operating was then adopted. Instead of passing the hydrogen through a red hot tube, it was burned in a small jet, over which a tube was inverted one foot long and ⅜ of an inch in diameter, so bent as to condense and retain all the moisture, the product of the combustion, with whatever else it might hold in solution. The same zinc that was used in the last experiment, remaining in the bottle and the acid and water as before, the combustion of the gas was continued for two hours, until two or three grammes of water were condensed. This fluid was poured into a watch-glass and allowed to evaporate spontaneously.

The liquids, which had been tested for arsenic in the gas-bottle before, were now introduced again, and the process conducted as in the last, till the gas-bottle had been emptied six times. The condensed water was poured into another watch-glass and set aside to evaporate.

Under a magnifier the latter watch-glass showed considerable crystaline matter—mostly beautiful prismatic needles, sometimes arranged in radiated groups. The incrustation in the first watch-glass presented no crystaline appearance of this distinctness or character.

*Tested by Ammonio-nitrate of Silver.*—A drop of distilled water was then placed in each glass, and, after the sediment was dissolved, a small portion of a drop of carefully prepared ammonia-nitrate of silver added to each. The contents of the first—that obtained by the combustion of gas from the zinc and acid—turned a little brown with no distinct precipitation or any further change. In the other glass there appeared instantly a lemon-yellow precipitate, exceedingly minute, but distinct and characteristic: like arsenite of silver; and resembling nothing else, except tribasic phosphate of silver, which could not be present under the conditions of the experiment. The precipitate soon became greenish-yellow, and then dark on standing. The attempt to reduce the arsenic from it to the metallic state failed; probably in consequence of the almost infinitesimal quantity on which the experiment had to be made.

*Examination of the Stomach itself.*—There remained still the stomach itself to be experimented on ; and it seemed desirable to make an examination of that with greater care and precaution than had been used in the preceding experiments. The old lot of sulphuric acid had become exhausted and some new was therefore procured. The gas-bottle was reconstructed, so as to be used more conveniently, and the capillary tube attached to it re-arranged, so that a portion of it, one to one and a half inches long, could be readily kept at a red heat over an alcohol lamp, for an indefinite time ; while the part beyond, sheltered from the heat by a perforated screen of brass foil, was carefully kept cool by a bit of moist paper.

*Purity of the Reagents.*—The first experiment was directed to ascertaining the purity of the reagents; and in this some fragments of common sheet zinc were employed. The passage of the gas was continued for about half an hour, the tube being red hot the whole time. Very soon the tube became brown at the distance of one fourth inch beyond the screen, and, at the close of the experiment, presented a fine irridescent steel-colored ring, brown on the edge farthest from the flame, and the whole about one fourth of an inch in length. The interior diameters of the tubes used in these investigations were between one-sixteenth and one-eighth of an inch. This ring readily disappeared on gently heating it over a lamp, condensing again, at a little distance, as a white sublimate, which, under a magnifier, showed the bright triangular faces of the crystals of arsenious acid. Some of the substances therefore used in this experiment contained arsenic.

The zinc was first rejected, and some new taken, which was supposed to be from the same pig, as that used in previous experiments. On passing the gas from this through the red hot tube, for one hour, a darkish spot made its appearance close to the screen of brass foil. On sealing the tube and heating, this appeared to melt and run together, without subliming. A very slight white cloud was seen, quite distant from the part heated, which on resubliming and examining under the microscope, was found to consist solely of little drops of moisture. In another part of the tube a very few scattered crystals were discovered, which seemed to be prismatic; but it was difficult to determine their form.

A few more pieces of zinc were then added to that already in the bottle, and acid being supplied from time to time, care was taken to evolve the gas more slowly, and not to pass it so rapidly through the tube. This was continued till the bottle had been emptied eleven or twelve times. The tube, sealed and examined under a magnifier, showed no dark stain. On heating, a very slight cloud, as of moisture, was seen, which on re-heating entirely disappeared, and nothing could be discovered in the whole tube, from one end to the other, when examined carefully with the highest powers of the microscope. These trials were regarded as sufficiently conclusive of the purity of all the materials used in the analysis.

*Solution of the Stomach.*—About two thirds of the stomach was cut into small pieces, and digested at a gentle heat, in pure distilled water. On straining it, while warm, a solution was obtained which slightly gelatinized on cooling. The gas-bottle being brought to a temperature a little elevated above that of the room, by standing in a basin of warm water, this decoction was introduced, and the process conducted as before, the same zinc remaining in the bottle. Even with the precaution of warming the bottle, the frothy bubbles were so tenacious that it was necessary to evolve the gas with exceeding slowness. The process was continued several hours, the gas-bottle being one-half emptied about ten times, making in all about two and one-half pints, passed through the tube. Beyond the heated portion, the tube became slightly stained very soon, and at the close of the experiment was lined with a brown ring, extending along its bore for the space of one-half inch; but continuous only near the flame, and in the part more distant consisting of scattered spots.

This tube, after being cut off and sealed at both ends, was gently heated on one side for an instant, in the flame of an alcohol lamp. The portion of the brown crust to which the heat was applied, instantly disappeared and immediately condensed again, presenting the appearance of a white cloud lining the tube, which, under a magnifier, was seen to consist of beautiful, lustrous, octahedral crystals.

*Repeated.*—This experiment was again repeated on the same materials, with the same result. It was also repeated on the same materials, with the addition of other matters before experimented on. The only thing worthy of note in the repetitions was, the apparent diminution in the quantity of arsenic with each successive trial. This result is entirely in accordance with comparative experiments made on liquids, to which minute portions of arsenic had been added.

In all these experiments there has been separated from the stomach and from the fluids in contact with it, a substance which could not be obtained from the reagents employed in the same process, and was, therefore, not contained in them, or only in proportions too minute to be detected. This substance cannot be other than arsenic.

*Not antimony.*—Antimony exhibits reactions which bear a closer resemblance to those described above, than any other substance. It is separated from its solutions in the same way, by nitrogen, and is again reduced by a red heat, to the metallic state. On heating this metal, with exposure to the air, it is converted into a white sublimate, oxide of antimony, isodimorphous with arsenous acid, and which consists sometimes of similar, bright, octohedral crystals. This change, however, is not effected at so low a temperature as with arsenic; the sublimation of the oxide of antimony also requires a much greater heat, and is not so entire and complete, because a part of it is apt to undergo a higher degree of oxidation, and in this state ceases to be volatile. But more than this, the metal itself is volatile, and is therefore not deposited beyond the heated portion of the tube, but in it: and the tube is blackened or browned at a different place from what it is if arsenic is present.

These points, conclusive enough in themselves, are still farther confirmed by the yellow precipitate, thrown down by ammonio-nitrate of silver, in the water, obtained from the combustion of the hydrogen. This, in itself, is diagnostic; for no such precipitate could have been obtained if the metal had been antimony.

*Conclusion.*—These experiments, therefore, were regarded as giving to the conclusion that arsenic was present a high degree of certainty. Had the poison been found in greater quantity, other tests might have been applied, and its existence might have been rendered more evident. Yet the reactions obtained are distinctive, and there is no reason to doubt or hesitate at the conclusion.

*Quantity.*—The amount of arsenic which was obtained is, indeed, frightfully minute. Trial experiments show that if arsenous acid constitutes but $\frac{2}{100,000}$ of the liquids in the gas bottle, the burning jet gives instantly a brown spot on porcelain, and, if held longer in the flame, the spot becomes bright and metallic, like polished steel. If the arsenic is in the proportion of $\frac{1}{100,000}$ or less, no stain makes its appearance. The proportion, therefore, of the contents of the bottle containing the stomach, which could have consisted of this poison, must have been less than $\frac{2}{100,000}$ or $\frac{1}{500}$ of 1 per cent. Other comparative experiments lead to the conclusion that it must have been in proportion greater than $\frac{1}{1,000,000}$. Suppose it $\frac{1}{500,000}$ and that the stomach and liquids with it weighed $2\frac{1}{2}$ pounds, avordupois, about 10,000 grains. This gives for the total amount of arsenic present $\frac{1}{50}$ of a grain! However strange such a conclusion may seem, it agrees well enough with the minuteness of the sublimates obtained in the various experiments.

*Source of the Arsenic.*—The quantity is so small that perhaps a greater uncertainty may arise as to its source. May it not have been arsenic acid, which, it has been supposed, may replace, in virtue of its isomorphism, the phosphoric acid in the animal structures; especially the phosphate of lime, by arseniate of lime in bone? That this ever takes place, may be regarded as still needing demonstration. It would seem to be an uncommon occurrence at best. The tissues, certainly, in which we might expect to detect it, would not be the soft ones; but those of bony consistence, which are composed, in large part, of phosphate of lime. The origin of the arsenic found here can hardly be ascribed to such a source.

*Does the quantity agree with the facts of the case.*—It may be more to the point to enquire if the results of the chemical examination are such as might have been expected from the character of the case, as it appeared in the testimony given before the court. The woman took the poison in a hearty meal; vomited constantly for forty-eight hours; and at this time, when first seen by a physician, was retching and vomiting every few minutes; this state of things being accompanied by an "inextinguishable thirst." After this she was cathartacised, and died six days or more from the commencement of her illness. Had arsenic in substance remained in the stomach, she could not, probably, have survived thus long.

Administered mixed with the food, most of it must have been thrown off with the first vomiting. It seems rather surprising that any trace of it should have remained, even in the tissues of the stomach, than that so little should have been found. This, at least, may be safely said, that in a case of such character, the small amount of arsenic detected is what might have been expected, and should be no matter of surprise or suspicion.

